# A study of rhinoplasty at a tertiary care institute in India

**DOI:** 10.6026/973206300211433

**Published:** 2025-06-30

**Authors:** Raghu Chanakya K.S., Lakshmi Sushma Vadali J., Monica M.

**Affiliations:** 1 Department of Otorhinolaryngology, GITAM Institute of Medical Sciences and Research, GITAM Deemed to be University, Visakhapatnam-530045, Andhra Pradesh, India; 2 Department of Plastic surgery, GITAM Institute of Medical Sciences and Research, GITAM Deemed to be University, Visakhapatnam-530045, Andhra Pradesh, India; 3 Department of Pathology, GITAM Institute of Medical Sciences and Research, GITAM Deemed to be University, Visakhapatnam-530045, Andhra Pradesh, India

**Keywords:** Nasal obstruction, rhinoplasty, DNS, septal cartilage

## Abstract

The ideal procedure to correct nasal obstruction in persons with external nasal deformity by comparing two techniques of rhinoplasty i.e. open technique and endoscopic technique is of interest. Hence, a prospective study was done for a period of one and half year and a total of 50 patients who underwent rhinoplasty procedures with various pathologies were evaluated. The most common pathology identified was deviated nasal septum (DNS) with external deformity and the most common symptom was nasal obstruction. Endonasal/closed technique was done in Group A and Group B patients underwent open rhinoplasty technique, with septal cartilage being the most commonly used graft in both the techniques. Both external and closed techniques are giving almost equal results in both functional and aesthetic aspects with minimal complications and the outcome was highly depended on the skillset of the surgeon.

## Background:

Rhinoplasty is indicated in deformities of nose for both functional and cosmetic purposes. Many persons with cosmetic nasal deformities are particularly conscious about their appearances and seek aesthetic improvement due to increasing social pressures and fast changing society patterns [1]. Rhinoplasty is one of the most commonly performed cosmetic surgeries and, in many cases, an elective procedure [2]. Therefore, it is crucial to assess the functional and aesthetic issues of the patient accurately and devise the proper surgical strategy most beneficial to the patient. Modern rhinoplasties can be approached by two distinct techniques: the open technique and the closed or endonasal technique [2]. Each rhinoplasty procedure, with its inherent strengths and weaknesses, has to be matched with a given nasal condition and to be used to get the desired results to offer the maximum advantage to the patient [1]. Therefore, it is of interest to report the ideal procedure to correct nasal obstruction with external nasal deformity by comparing the procedures i.e. open technique of rhinoplasty and closed technique of rhinoplasty and to evaluate the complications in both groups.

## Materials and Methods:

## Data source:

The present study was conducted in a Tertiary care hospital, Visakhapatnam for a period of 18 months.

## Study design:

It is an analytical prospective study.

## Study population:

Total 50 patients with deformities of nose, having nasal obstruction, who fulfilled the inclusion criteria, were taken in the present study. The total patients were assigned into 2 groups with 25 each based on the approach whether it is a closed or external.

## Group A:

This group includes 25 patients who underwent closed/ endonasal rhinoplasty addressing both the functional and aesthetic components.

## Group B:

This group study includes of 25 patients who underwent external/open rhinoplasty addressing both the functional and aesthetic components.

## Inclusion criteria:

[1] Deviated nasal septum with external deviation

[2] Nasal bone fractures

[3] Saddle nose deformity

[4] Hump nose

[5] Tip deformities

[6] Crooked nose

[7] Alar flaring

[8] Patients above 16 and below 60 years of age

## Exclusion criteria:

[1] Patients associated with nasal polyposis, tumours, granulomatous diseases and malignancies of nose and para nasal sinuses

[2] Patients with acute rhinitis

[3] Patients below 16 and above 60 years of age

## Methodology:

The study includes the age and gender variations of the patients who underwent rhinoplasty for various deformities of the nose satisfying the inclusion criteria. The ideal procedure for those patients was identified and the different approaches of rhinoplasty and their complications were evaluated. The preoperative evaluation of the nose is done carefully. In all the patients complete clinical examination was done. The severity of nasal obstruction was assessed subjectively. Bimanual digital examination determining the size, shape and thickness of lower lateral cartilage and columella was done. In all the patient's complete history was taken and clinical examination was done. Procedure and follow up is done as per the standard protocols.

## Results:

In Group A out of 25 patients, 14 were males and 11 were females who underwent closed/endonasal rhinoplasty whereas, in Group B out of 25 patients, 12 were males and 13 were female patients underwent open/external rhinoplasty. The overall study group was a male predominant (Table 1 - see PDF and Figure 1 - see PDF). Most of the patients in the study group belonging to 21- 30 years of age that is 30 patients (60%) (Table 2 - see PDF and Figure 2 - see PDF). In the Group-A who underwent closed rhinoplasty, majority of the patients 16 (64%) were in the age group 20-30 years followed by 5 (20%) patients were in the age group of 11-20 years. In the group - B who underwent open rhinoplasty majority of the patients 14 (56%) were in the age group 20-30 years followed by 5(20%) patients were in the age group of 30-40 years. In the total study population, 20-30 age groups underwent rhinoplasty most commonly (Table 2 - see PDF and Figure 2 - see PDF). Most common pathology addressed is Deviated Nasal Septum (DNS) with external deformity (Table 3 - see PDF and Figure 3 - see PDF). In the cases who underwent closed rhinoplasty, 18 (72%) patients were having DNS with external deformity, 2(8%) were with saddle nose, 2 (8%) were having hump nose, 1 (4%) patient was having tip deformity and 2(8%) patients were with crooked nose. In the cases who underwent open rhinoplasty, 13 (52%) patients were having DNS with external deformity, 4 (16%) were with saddle nose, 2 (8%) were having hump nose, 1 (4%) patient was having tip deformity and 5 (20%) patients were with crooked nose (Table 3 - see PDF and Figure 3 - see PDF). Septal cartilage was found to be most commonly used graft material in both open and closed rhinoplasty (Table 4 - see PDF and Figure 4 - see PDF). In cases that underwent closed type of rhinoplasty, 14 (56%) cases septal cartilage and in 8(32%) cases conchal cartilage and in 3 (12%) cases rib cartilage was used as a graft/implant. In cases who underwent open type of rhinoplasty- in 12(48%) cases septal cartilage and in 6 (24%) cases conchal cartilage and in 7 (28%) cases rib cartilage was used as a graft/implant (Table 4 - see PDF and Figure 4 - see PDF). Out of 50 patients in the study, 31 patients were found to have associated nasal obstruction, of which 18 patients underwent closed rhinoplasty and remaining 13 underwent open rhinoplasty (Table 5 - see PDF and Figure 5 - see PDF). Out of 31 patients, closed rhinoplasty was done in 18 patients with nasal obstruction, in follow up period all the patients were relieved of nasal obstruction and in remaining 13 patients open rhinoplasty was done and after 3 months of follow up all the 13 patients were comfortable and no complaints of nasal obstruction (Table 5 - see PDF and Figure 5 - see PDF). Excessive bleeding was found to be most common complication in both closed (16%) and open (12%) rhinoplasty (Table 6 - see PDF and Figure 6 - see PDF). In the cases who underwent closed rhinoplasty, 2 (8%) of patients had excessive bleeding, 1 (4%) of patients had ecchymosis and edema, 2(8%) had persistent minor deformities, 2 (8%) of patients had synechiae and 1 (4%) had infection. In the cases who underwent open rhinoplasty, 1 (4%) of patients had excessive bleeding, 1 (4%) of patients had ecchymosis and edema, 1(8%) had persistent minor deformities, 1 (4%) of patients had synechiae and 2 (8%) had infection (Table 6 - see PDF and Figure 6 - see PDF).

## Discussion:

As Rhinoplasty is indicated for both functional and cosmetic deformities of nose this study was done to compare the outcomes of both open and closed techniques of rhinoplasty to analyse which technique gives the maximum benefit to the patient with minimal complications. The present study consists of 50 patients, 26(52%) males and 24(48%) females. In Group A out of 25 patients, 14 (56%) were male and 11(44%) were female patients, where as in Group B includes 12(48%) males and 13(52%) female patients. The male to female ratio of the present total study group was 1.08:1. This study aligns with study done by Biggs *et al.* [3] in rhinoplasty surgery on 100 patients in which 52 patients (52%) were males and 48(48%) were females with a ratio of 1.08:1. Spataro *et al.* conducted a retrospective cohort study on Revision rates and Risk factors of patients undergoing rhinoplasty in which 57% were males and 43% were females with a ratio of 1.32:1 [4]. In the observations of Randhawa *et al.* (2016) a prospective cohort study on outcome of rhinoplasty surgery on olfactory function, out of 43 patients the sex incidence was 26 (60%) males and 17 (40%) females with a ratio of 1.53:1 [5]. The observations of the present study were almost comparable with Biggs *et al.* [1], Spataro *et al.* [4] and Randhawa *et al.* [5]. Overall, the study group was a male predominant. This can be attributed to more exposure to trauma in males or random assignment of patient. In the present study population ranged from 11 to 60 years with mean age of 25.42 years. Most of the patients in the study group belonging to 21- 30 years of age that is 30 patients (60%). Our observations are different from that of study done by Biggs *et al.* [3], Spataro *et al.* [4] and Randhawa *et al.* [5] where the mean age was 36 years, 41.0 with SD of 15.3 years and 34.1+/- 12.2 years respectively. The variations in mean age observed in various studies were due to the difference in age distribution and study population size. In our study population of 50 patients, a total of 31 cases were having DNS with external deformity out of which 18 patients had undergone closed rhinoplasty and 13 had open rhinoplasty. The present study included 7 cases of crooked nose deformity of which 2 cases with minor deformity, closed rhinoplasty were done and 5 cases with gross deformity we preferred open rhinoplasty. The study encountered 6 cases of saddle nose deformity, among which 4 patients having gross deformity underwent external rhinoplasty; the remaining 2 underwent closed rhinoplasty. Out of 4 hump nose cases, we did closed rhinoplasty in 2 subjects and open rhinoplasty in 2 subjects. In 2 patients with tip deformity, one each had undergone open and closed rhinoplasty. For achieving successful correction of the deviated nose all anatomic components involved in the deformity have to be recognized and treated accordingly. Septal surgery plays a crucial role in the management of the deviated nose. Even in the absence of functional problems, correction of minor and major septal deviations can determine realignment of the external nose. The type of approach is determined by detailed preoperative evaluation of the patient and in patients with simple/minor deformity we preferred closed approach and alternatively, an open approach is indicated for treating a twisted nose, heavy deformities and S-shaped deviations. So the selection of type of surgery was done purely on the basis of patient requirement and type of deformity dealt with. The study findings were consistent with study done by Parrilla *et al.* [6] on septorhinoplasty in DNS. In 25 patients with closed rhinoplasty, septal cartilage was used in 14 cases, conchal cartilage in 8 patients and rib cartilage in 3 patients. In the remaining 25 patients with open rhinoplasty, septal cartilage was used in 12 cases, conchal cartilage in 6 patients and rib cartilage in 7 patients. As the goal of septorhinoplasty is reconstruction of the nasal skeleton to provide adequate structural support allowing for optimum functioning of the nasal airway and achieving an aesthetically pleasing result, the current study used autogenous grafts particularly cartilaginous types, largely because of their good acceptance rate, durability, virtual lack of an immunogenic response, low infection and extrusion rates when compared with others [7, 8- 9]. The septal cartilage was most commonly used as it was easily accessible from the same site of surgery and conchal cartilage was used in patients with minor deformity where there is a compromise of septal cartilage. We had used rib grafts in gross deformities and in patients where there is no adequate septal or conchal cartilage. Autogenous cartilage grafting offers strength for nasal support to replace or augment missing tissue with similar tissue and recreates the nasal anatomy as close to normal as possible. In a study conducted by Moretti *et al.* [10] Grafts in septorhinoplasty, it was mentioned that autografts do not induce an immune response, and also have the lowest infection and extrusion rates of any currently available materials. Rib cartilage grafts are the gold standard for nasal reconstruction in patients with cartilage depletion and when large amounts of tissue are needed and recreate the nasal anatomy as close to normal as possible. In the cases who underwent closed rhinoplasty, 2(8%) of patients had excessive bleeding, 1(4%) of patients had ecchymosis and edema, 2(8%) had perisistent minor deformities, 2(8%) of patients had synechiae and 1(4%) had infection. In the cases who underwent open rhinoplasty, 1(4%) of patients had excessive bleeding, 1(4%) of patients had ecchymosis and edema, 1(4%) had perisistent minor deformities, 1(4%) of patients had synechiae and 2(8%) had infection. In our study, in patients who underwent closed rhinoplasty the complications like excessive bleeding, synechiae and persistence of minor deformities were more when compared to open rhinoplasty. The complications like infection were more common in patients with open rhinoplasty. Durfishan *et al.* [11] conducted a study on satisfaction and outcome after augmentation rhinoplasty in 56 patients at a tertiary care hospital and found that augmentation rhinoplasty can provide satisfactory aesthetic and functional outcomes for patients with nose deformities with consistent improvement across all domains related to aesthetics and functionality. Neaman *et al.* [12] conducted a study on cosmetic rhinoplasty in 369 patients in which overall complications reported were 7.9% of which infection was developed in 6 patients, post-operative edema in 3 patients and hemorrhage in 3 patients and ecchymosis in 2 patients and other complications in 7 patients.

## Conclusion:

We show that both closed/endonasal rhinoplasty and open/external rhinoplasty are having their own merits and demerits and surgical approach has to be personalised based on complexity of the deformity, aesthetic goals and patient's anatomy. Both external and closed are giving almost equal results in both functional and aesthetic aspects. Both procedures are having minimal complications with negligible variations.

## Funds and Support:

Nil
